# The transformation of provisioning systems from an integrated perspective of social metabolism and political economy: a conceptual framework

**DOI:** 10.1007/s11625-021-00952-9

**Published:** 2021-04-18

**Authors:** Anke Schaffartzik, Melanie Pichler, Eric Pineault, Dominik Wiedenhofer, Robert Gross, Helmut Haberl

**Affiliations:** 1 Institut de Ciència i Tecnologia Ambientals, Universitat Autònoma de Barcelona (ICTA-UAB), Barcelona, Spain; 2 Institute of Social Ecology (SEC), University of Natural Resources and Life Sciences (BOKU), Schottenfeldgasse 29, 1070 Vienna, Austria; 3 Institut des Sciences de l’Environnement, Université du Québec à Montréal (UQAM), Montreal, Canada; 4 Institute of History and European Ethnology, University of Innsbruck, Innsbruck, Austria

**Keywords:** Capitalist provisioning, Material stocks, Capital, Fossil energy system, Distribution, Socio-ecological conflicts

## Abstract

Energy, food, or mobility can be conceptualized as provisioning systems which are decisive to sustainability transformations in how they shape resource use and because of emissions resulting from them. To curb environmental pressures and improve societal well-being, fundamental changes to existing provisioning systems are necessary. In this article, we propose that provisioning systems be conceptualized as featuring integrated socio-metabolic and political-economic dimensions. In socio-metabolic terms, material stocks—buildings, infrastructures, and machines, for example—are key components of provisioning systems and transform flows of energy and materials into goods and services. In political-economic terms, provisioning systems are formed by actors, institutions, and capital. We loosely identify and closely analyze, from socio-metabolic and political-economic perspectives, five phases along which provisioning systems are shaped and in which specific opportunities for interventions exist. Relying mainly on examples from the fossil-fueled electricity system, we argue that an integrated conceptualization of provisioning systems can advance understanding of these systems in two essential ways: by (1) facilitating a more encompassing perspective on current forms of provisioning as relying on capitalist regulation and on material stocks and flows and by (2) embedding provisioning systems within their historical context, making it possible to conceive of more sustainable and just forms of provisioning under (radically) altered conditions.

## Introduction

The notion of provisioning systems provides a conceptual lens to analyze how goods and services sustaining individuals and their societal mode of living are generated and reproduced. Rooted in heterodox economics (Fine 2002; Jo et al. 2017), provisioning systems—for energy, food, or mobility, for example—are understood as linking resource use and social outcomes (O’Neill et al. 2018; Fanning et al. 2020). As such, the concept allows for the integrated consideration of the material and political-economic dimensions of provisioning systems, that is, for the analysis of institutions, actors and capital flows and how they drive and interact with stocks and flows of energy and materials. Such an approach is prerequisite to transforming these systems, to achieving more equitable and just access to the commodities and services they yield, and to curbing resource use and the generation of wastes and emissions globally.

To understand how provisioning systems transform flows of energy and materials into goods and services, we conceptualize and investigate them with a particular focus on their socio-metabolic dimensions. As is true of socio-economic systems more generally, provisioning systems rely on resource in- and outputs, transformation and stockpiling and are shaped by such flows and stocks (Fischer-Kowalski and Erb 2016). We integrate this socio-metabolic perspective with one that focuses on the political-economic institutions, actors and their power relations in provisioning systems (Monstadt 2009; Bayliss et al. 2013). These are, of course, interrelated with and functionally dependent on what social metabolism defines as material stocks: the infrastructure, buildings, and machinery to which accumulated resource inputs give rise over time. Conceptualizing provisioning systems in interrelated socio-metabolic and political-economic terms is key in identifying the challenges to and possible entry points and strategies for sustainability transformations.

In their current expressions, provisioning systems are strongly shaped by capitalist relations (cf. Lee 2012), which is simultaneously an indication of their context dependence (Jo et al. 2017). Under other conditions and circumstances, other forms of provisioning than those currently in place are conceivable. Because of their capitalist configuration, the power relations between actors involved and their vested interests, and the sheer force of their materiality, provisioning systems have a crucial impact on the *distribution* of resource flows, that is, on who has access to the goods and services they provide (Swilling 2011). The construction of infrastructure lays claim to land, binds material resources, and embodies energy; their subsequent operation requires further resource inputs and also generates and distributes material and energy outputs (alongside with wastes and emissions). By failing to consider the effect of the socio-metabolic dimensions of the provisioning system, one could easily arrive at the conclusion that transformation is a matter of the insight and the will of the involved actors. And while that is undoubtedly important, its lack is not the only obstacle to transformation.

**Fig. 1:**
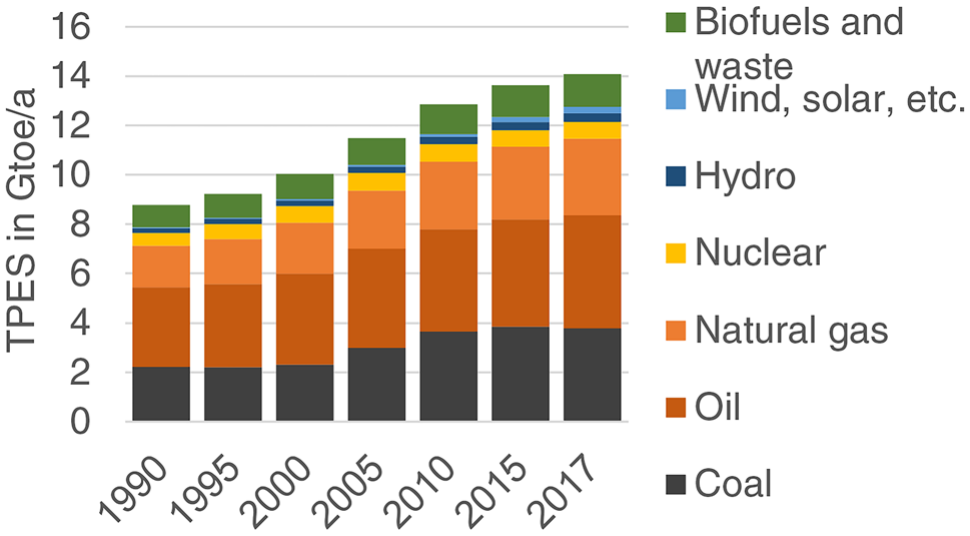
World total primary energy supply (TPES) by source in Gigatons (109 tons) of oil equivalent per year (Gtoe/a), 1990–2017; source of data: IEA (2019a)

We root our conceptual proposal in examples from the global energy system, which is “the largest network of infrastructure ever built” (Seto et al. 2016, p. 426). From coal mines and oil rigs to power plants and electric grids, the fossil energy system relies heavily on material stocks, both within extraction and production and in linking to final consumption. The exploitation of fracked gas and oil and tar sands, for example, is currently creating a wave of new infrastructures. Rather than abandoning fossil fuels as the dominant source of energy as one of the requirements of a sustainability transformation, the currently ongoing energy transition is solidifying, not challenging, the global fossil energy system (Schaffartzik and Fischer-Kowalski 2018). Fossil fuels contributed 81% to global primary energy in 2017 (oil 32%, natural gas 22%, coal 27%), while the shares of hydroelectricity (3%), nuclear (5%) and renewables (11%) were modest (Fig. 1). Electricity generation and use rely heavily on fossil fuels (38% coal, 23% natural gas) with hydropower and renewables only accounting for 25% of generation (IEA 2019a). But the electricity system is also treated as a key piece in the sustainability transformation, e.g., through electricity generation from renewable resources or by replacing oil through electric mobility. In this perspective, the transformation of the energy system is debated in terms of energy demand and technological constraints, neglecting the political economy of actors and institutions that control transformative change (Moe 2017).

In this article, we develop a framework for analyzing the integrated socio-metabolic and political-economic dimensions of provisioning systems. Using the example of the fossil energy system, we identify five phases in which provisioning systems are shaped and in which specific opportunities for interventions exist. We fill a gap between analyses focusing on the socio-metabolic and techno-material properties of provisioning systems but ignoring their societal regulation (Haberl et al. 2019) and those analyses that consider institutions and/or actors but not the materiality of these systems (Breetz 2017). We arrive at an understanding of these systems that simultaneously builds on previous work on social provisioning (e.g., Fine 2002; Jo et al. 2017) and challenges this understanding in two ways. Namely, by arguing that the outcomes obtained through provisioning processes are diverse and that provisioning systems are not necessarily geared towards providing potential societal benefits, such as energy or food or mobility, or towards keeping the associated levels of resource use and emissions low. In addition, we demonstrate that material and energy stocks must essentially be considered in the conceptualization of provisioning systems but tend to only be marginally touched upon (a research gap exposed by, for example, Ekeland and Sœther 2017; Jo and Todorova 2017).

The article proceeds as follows: in “Provisioning systems from a socio-metabolic and political-economic perspective”, we present how we employ the provisioning systems approach. In “(Re)shaping provisioning systems: phases and their possibilities for intervention”, we apply this approach, using the example of the energy system and identifying five phases in the (re)production of provisioning systems, from access to land for infrastructure development to the possible dissolution of existing stocks. During each phase, conflicts, resistance, and counter-claims offer opportunities for interventions and entry points for the transformation of the fossil energy system.

## Provisioning systems from a socio-metabolic and political-economic perspective

We make a case for an integrated consideration of social-metabolic and political-economic dimensions of provisioning systems, building upon existing research on social provisioning.

### What are provisioning systems?

Rather than accepting an understanding of economics as the science of managing scarce resources, some heterodox economists have insisted that economics studies those processes that represent social provisioning (Lee 2012; Jo et al. 2017). These processes range from “the process whereby surpluses are created in economies, how they are extracted, who gets them and what they do with them” (Harcourt 1986, p. 5) to “the process that provides the flow of goods and services required by society to meet the needs of those who participate in its activities” (Lee 2005, p. 30).

Social provisioning refers to “use-value of goods and services” and is often thought to represent processes that—in contrast to the provisioning of “exchange-value or monetary profits” (Jo and Todorova 2017, p. 33) or “pecuniary gain”—are necessary and must be sustained (Henry 2017, pp. 166–168). Whether a provisioning process is approached as primarily generating use value or exchange value reflects the underlying understanding of (economic) reproduction and has far-reaching impacts in terms of the actors, institutions, and power relations considered (or not) in the analysis of these processes. We take the use value as our point of departure (energy transformed and distributed in the energy system, for example), recognizing provisioning as influenced by “technology, employment, income, and welfare” (Jo and Todorova 2017, p. 35), with each particular manifestation of social provisioning dependent on cultural, economic, and historical context (Lee 2012; Jo et al. 2017). In capitalist forms of provisioning, monetary or power gains can clearly be the criteria according to which processes are organized, having greater relevance in decision-making than the effective production and just distribution of use value. Capitalist forms of provisioning are distinct from other forms, because goods and services take the form of commodities, implying according institutional arrangements and relationships (Jo et al. 2017, pp. 16–17).

Considering the economy in terms of systems of provisioning constitutes a productive analytical alternative to the oversimplified intersections of supply and demand curves. As an approach to understanding the link between production and consumption (Fine 2002, p. 80), or, put differently, resource use and social outcomes (O’Neill et al. 2018; Fanning et al. 2020), the notion of provisioning systems is sufficiently broad to allow for specification depending on the research question. While the need to distinguish between different provisioning systems has been strongly represented in some of the seminal conceptual work (Fine 2002), our research is particularly interested in the areas of overlap, in the sometimes difficult distinction between the different forms of provisioning. Decentralized, locally based provisioning of renewable energy (e.g. through roof-top solar panels) could be considered a system distinct from large-scale energy generation and distribution (e.g. thermal power plants and electric grids). However, decentralized electricity provisioning also has a bridging function with regard to the main grid, enabling the use of electric appliances and generating demand for electricity and the (eventual) connection to the main grid. This approach allows us to entertain the possibility of the renewable system forming part of an over-arching fossil-based system.

### Provisioning energy and electricity

In this article, we will draw examples from energy and, in particular, electricity provisioning to illustrate the implications of an integrated socio-metabolic and political-economic perspective. Socio-metabolic analyses of societal energy transitions provide an elaboration of the notion of provisioning systems as dynamic, historical, and dependent on societal context: from the passive use of solar radiation through hunting and gathering to its controlled use in agriculture and finally, the reliance on fossil fuels and hence on biomass produced (through solar radiation) millions of years ago (Fischer-Kowalski and Schaffartzik 2015), “the methods available for converting solar radiation into useful energy” (Ekeland and Sœther 2017, p. 414) have formed and continue to form the foundation for any kind of provisioning. The associated changes have been especially radical and far-reaching when societies began to use solar radiation of the past (fossil fuels) at a larger scale (Fischer-Kowalski et al. 2018) and we focus on the current state and trajectory of the fossil energy system. In better understanding provisioning systems, resource flows and stocks in general and those related to energy in particular belong front and center.

Energy provisioning is strongly shaped by the materiality of the infrastructures through which energy is captured and transformed into services for consumption. This is true to the extent that the current fossil energy system would continue to shape resource flows even if a globally concerted commitment to rapid phase-out could be reached. Individualized transport and the drivers (in the figurative sense!) which led to its rise are among the central tenets of the fossil energy system; to the extent, that even in the context of transformation, debates are over replacing combustion motors with electric ones rather than reimagining public transport. But even true transformation towards sustainable, shared infrastructures would, to begin with, generate high energy demand as well as wastes, making the legacy effect of the fossil energy system felt. Well over 1 Gt of metals, plastics, glass, and rubber would have to be disassembled and recycled or landfilled, with dismantling alone causing greenhouse gas emissions in the order to 35 Mt CO_2_ equivalent (1 Mt = 10^6^ metric tons) to get rid of the global fleet of 947 million passenger cars in use in 2015 (Nakano and Shibahara 2017; OICA 2019). Of course, fossil-fuel infrastructures and technologies that must be phased-out in sustainability transformations (Heyen et al. 2017) continue to determine patterns of resource use and emissions while they are in use. Simply by continuing to use the fossil-fueled structures that have already been built, the annual greenhouse gas emissions in 2030 will be twice as high as the amount that would make it realistically possible to limit global heating to 1.5 °C (UNEP 2019).

On the consumption end, energy usually appears as embodied into a good or a service. In final consumption, electricity is not only an important form in which energy is made available but also one that is—according to some currently dominant approaches—to play a crucial role in ‘greening the economy’. In the electricity provisioning system, services such as light, cooling/heating, communication and entertainment, and, to an increasing extent, mobility are consumed (Cullen and Allwood 2010), allowing utilities companies to sell electricity. But even on this ‘last leg’ of the provisioning process, it is a wider system that we must understand to understand electricity provisioning. Although the focus is often on them, neither the consumers (e.g., households) nor the suppliers (utilities companies) are equipped to address the efficiency shortcomings in the energy system. Instead, engineers and researchers, appliance manufacturers, advertising, government regulatory bodies and many other actors and institutions play a pivotal role in defining the framework within which provisioning must occur. Sustainable and just electricity and energy systems require a departure from current forms of provisioning. Interventions such as legislation on some of the emissions from coal combustion, mandatory energy efficiency labeling for housing and appliances, or the ban on incandescent light bulbs, will have a role to play but, on their own, are no match for the dimensions of the energy system. Transformations required to adequately address climate change (IPCC 2018) or the exhaustibility of fossil-fuel resources (GEA 2012) are not only techno-managerial fixes but political interventions (Meadowcroft 2011; Goldthau and Sovacool 2012; Brand 2016; Görg et al. 2017; Pichler et al. 2017). This is not to discredit interventions into the materiality of provisioning systems: in fact, political interventions that fail to acknowledge and address the role of material stocks and flows in shaping how provisioning works will ultimately not be successful in transforming these systems.

### Stocks and flows: socio-metabolic perspectives on provisioning

We concur with the research treating provisioning systems as the historical results of hinged socio-metabolic and political-economic processes (Heynen et al. 2006, p. 5; Swyngedouw 2009; Guy 2011). We operationalize the concept of social metabolism as encompassing the material and energy inputs, their transformation (for example, goods and services), the accumulation and reproduction of materials stocks, and all resulting outputs, involved in societal reproduction (Fischer-Kowalski and Erb 2016). As such, the concept of social metabolism is based on an understanding of societies not as purely social but as consisting of hybrid components that are also biophysical (Fischer-Kowalski and Weisz 1999). We understand that societies must reproduce themselves both socially and biophysically and that social organization shapes social metabolism just as much as social metabolism shapes organization. Provisioning systems are not only processes between production and consumption but include both of these activities, encompassing the actors and their relations previously referenced alongside tangible biophysical structures and processes. An understanding highly compatible to what we sketch out here has been applied to, for example, energy infrastructures (Bridge et al. 2018).

Growth in and distribution of resource extraction and consumption are powerful forces in the ongoing socio-ecological crisis. The concept of provisioning systems is a productive approach to investigating the links between production and consumption *and* the overall organization of society’s metabolism. This is crucial in understanding the socio-metabolic prerequisites to production and consumption and thereby also in coming to terms with their socio-ecological impacts. In doing so, we aim to elucidate critical drivers and determinants that are often not adequately considered.

Although the materiality of resources is mentioned in some of the provisioning system literature (see “What are provisioning systems?”.), they are not subject to the same rigorous analysis that the socio-economic dimensions of the systems are. The environment and the resources it harbors tend to provide the context within which provisioning systems operate without being operationalized as a component of the system (Lee 2012; Ekeland and Sœther 2017) or are passively affected by human agency. We make the case that conceptual depth and greater applicability stand to be gained from considering the role of material stocks and flows in structuring not just the provisioning system but also the wider economic system. This includes the form of resource use for socio-economic purposes and outcomes thereby obtained, such as control over land and people or the generation and protection of monopolies, for example.

Many existing applications of the provisioning systems concept point to the importance of the biophysical materiality of provisioning processes (Swilling 2011; Mattioli et al. 2020; Fanning et al. 2020), inviting further and more systematic conceptual integration.

The materiality of provisioning systems has long-lasting effects on overall patterns of resource use, with construction and building decisions made decades and even centuries ago partially determining which resources are used and where, today (Krausmann et al. 2017). At the beginning of the twentieth century, more than 75% of all material resources extracted globally were used dissipatively, consumed as food for humans, as feed for animals, or for technical energy in the combustion of fuel wood and fossil energy carriers. The remaining 25% of global resource use were dedicated to expanding and maintaining material stocks of infrastructure, buildings and machinery (Krausmann et al. 2018). Since then, the use of stock-building materials accelerated rapidly. Reconstruction in Europe and Japan after World War II, postwar economic boom, and urbanization in the industrialized countries coincided with average growth rates for stock-building materials of 4% per year from 1950 to 1980 (Krausmann et al. 2017, p. 1882). The share of dissipatively used material decreased to 40% by 2015, while their extraction surged in absolute terms to 36 Gt/a. At this point, 961 Gt of materials had been accumulated in stocks, averaging to 131 tons per capita of the world population (Krausmann et al. 2018). Not only were materials increasingly used to build-up stocks, but also the materials with dissipative uses also increasingly flowed *through* stocks. These materials were harvested or extracted by machinery, processed in factories, transported over long distances on roads or railway tracks, through pipelines or in cargo ships, stored, sold in buildings constructed specifically for retail, and manipulated with machines and appliances during consumption and use. Globally, societies mainly extract materials that can only be reached and used in significant amounts with the help of machines: more than 70% of resource extraction are abiotic materials: non-metallic minerals (i.e., mainly construction minerals), fossil energy carriers, and metals (Krausmann et al. 2018). The remaining 30% of extraction is largely industrially harvested and processed biomass, also requiring material stocks.

Material stocks correspond to the physical, tangible components of provisioning systems: the gravel and concrete amassed in a road, the iron and cement in a power plant and the metals and construction minerals in electricity grids. As such, stocks can be studied in terms of their role in society’s metabolism, through economy-wide material and energy flow accounting (Pauliuk and Müller 2014; Krausmann et al. 2017; Wiedenhofer et al. 2019). Social metabolism considers the systemic interrelations between the biophysical *flows* and *stocks* which society requires to reproduce itself (Haberl et al. 2019). The flows encompass material and energy inputs from the environment and from other economies (imports) and outputs to other economies (exports) and to the environment (wastes and emissions). Stocks include humans, their livestock and artifacts (buildings, machinery, and durable consumer goods).

Material stocks play a crucial role in locking societies into specific patterns of resource use (Unruh 2000; Seto et al. 2016). They constrict the socio-metabolic corridors, those spaces of resource use within which societal reproduction can occur. As stocks are amassed, often pairing new opportunities for consumption with new input requirements, societal option spaces to shape resource consumption, wastes and emissions dwindle. Natural gas, for example, was traditionally transported and distributed through dedicated, inflexible pipeline systems connecting sources with final users. Liquid natural gas (LNG) as a novel and burgeoning means of gas distribution is based on compression and cooling of gas into a liquid for transport. This allows for a wider range of transport options, at the expense of greater infrastructure (and hence material and energy) prerequisites. In addition to these pipeline systems, LNG requires liquefaction terminals and deep-sea ports, large and specialized gas tanker ships, and, finally, regasification plants, linked to gas distribution systems (IGU 2019).

Yet, it is not only the build-up but also the use of stocks that requires resources (Krausmann et al. 2017). Even though it revolves around large-scale stocks such as thermal power plants and electric grids, much of the impact of the fossil energy system is related to the flows (e.g., coal, oil, gas) required in the use of these stocks. For many forms of renewable energy, the reverse is true (Watari et al. 2019). Accumulated material stocks shape future resource use, enabling or requiring uses of certain materials or energies, while restricting societies’ options to alter their resource use patterns. The fossil energy system, with its centralized, single-function infrastructures, comprises a colossal and, in terms of alternative, post-fossil uses, inflexible system, mirrored by similar inert institutional structures. More than 650 Gt of CO_2_ will be emitted if all currently existing fossil-fueled stocks, that is, power plants, machines, automobiles, etc. are used until the end of their planned lifetimes. This alone would exceed the 420–580 Gt of CO_2_ emissions which may still be emitted if global warming is to be limited to 1.5 °C (IPCC 2018; Tong et al. 2019). All planned fossil-fueled power plants would additionally emit almost 190 Gt of CO_2_, raising emissions to a total of approximately 840 Gt, i.e. double the lower bound of the 1.5° emission budget (UNEP 2019; Tong et al. 2019).

In socio-metabolic terms, the formidable growth trajectory of the global economy (Steffen et al. 2015) has been based not only on increasing extraction but also on trade of material resources (Schaffartzik et al. 2014). The resulting metabolic inequalities (Duro et al. 2018) are also associated with an unequal spatial distribution of stocks. An accumulation of stocks both precedes and follows the expansion of material extraction and production into peripheral regions. Roads, for example, are often the first prerequisite to the stock-building in the establishment of agriculture or industry (Ciccantell and Bunker 1998; Laurance et al. 2015). A fossil-based energy system and the distribution of electricity is the prerequisite to further stock-building to such an extent that high investments and high CO_2_ emissions tend to coincide (Grimes and Kentor 2003). International patterns of stock-building play a pivotal role in differentiating spaces, e.g., by claiming them for production, by connecting or *not* connecting them to provisioning systems, and intensifying their connections through trade and delivery of products and services. These activities often materially manifest as land-use change (e.g., through urbanization) and are politically and economically accompanied or enforced by incentives for specific types of land use and barriers for others, legislation on land rights, or even (violent) dispossession.

### Capital, actors, and power relations: political-economic perspectives on provisioning

Contemporary (fossil-fueled) provisioning systems rely on capitalist regulation. Although capitalist forms of provisioning are discernible as such—as a result of their function to maximize profit, for example—this does not mean that they are all the same. Considering *regulation* (Lipietz 1987) emphasizes that provisioning systems are formed by a certain institutional constellation evolving from structural conditions, system dynamics, and power relations (Jessop 2002; Görg 2003). Within the capitalist regulation of provisioning, a certain accumulation regime can be linked to a certain mode of regulation (Lipietz 1987; Boyer 1990; Jessop and Sum 2006): accumulation regimes may differ depending on export orientation or the role of productive or financial capital in provisioning systems, for example. Modes of regulation may differ depending on institutional setups (e.g., more or less state involvement in provisioning, participation of unions or other civil society actors). The analysis of accumulation regimes and modes of regulation is helpful in better understanding the political economy of provisioning systems.

Apart from the more structural–institutional analysis provided by the regulation approach, an *actor-oriented perspective* helps in identifying the conscious and interest-driven decisions that shape these systems and the respective material stocks and flows. Actors engage in behaviors and practices as important constituting elements of provisioning systems (Shove and Walker 2014; Shove et al. 2015; Seto et al. 2016) and may comprise individuals but more often collective actors such as companies, business associations, international financial institutions, unions, social movements, policy-makers, and bureaucracies. Actors form coalitions or networks which shape and influence the institutions governing provisioning systems (Seto et al. 2016, p. 434) and ‘following the actors’ is an important approach to understanding inertia or potential for transformative change (van der Vleuten 2019). The state plays a central role in enabling not only the development of the provisioning system but also in facilitating or hindering the involvement of other actors. It is within the institutions of the state that not only decision-making but also coalition-building amongst actors with similar vested interests takes place (Moe 2017). However, global neoliberal restructuring since the 1980s has seen greater decision-making power over the structuring of provisioning systems allocated to markets and private corporations. The distinction between private and public (Fine 2002) or market, state, household, or communal modes of provision (Southerton et al. 2004), however, reflects the dominance rather than the exclusive involvement of one of these actors.

Power is mainly established, maintained and possibly overturned through the property relations of (material) stocks, defining access to and exclusion from provisioned goods and services. For example, in capitalist provisioning, if material stocks are a corporation’s assets, they are subject to the imperatives of accumulation and profitability. Public property may be organized according to the same principles. This can be observed, for example, in that many utilities are held publicly but sell electricity at a profit (Willis and Philipson 2018). Public property can also be not-for-profit or property can be held by cooperatives, with vastly different implications for the power relations in the provisioning system.

Power relations are also shaped by the organization of investments in the provisioning system. States, banks, private investors, and increasingly also (energy) cooperatives or individuals play a role in contributing the capital required to establish or maintain the provisioning system. These actors thereby wield influence over what is constructed and (eventually) destroyed. Capitalist power over assets typically takes three forms (Carroll and Sapinski 2018), all corresponding to ways through which ownership of and control over material stocks are mediated:

1. Corporations exercise direct operational power over their fixed capital assets by putting them to work from which they obtain operational profits,
2. They wield strategic power by actively managing the market and regulatory context in which they operate through research and development and innovation, in competition, through upstream suppliers and downstream costumers, and through lobbying regulators and policy-makers, and
3. They have allocative power through the profits they capture, the funds they can borrow from banks or on markets, and the structuring investments they make. These include not only the construction of new fixed capital assets, but also buying and selling of pre-existing fixed capital, and investment in the property of competing corporations to secure control.

**Fig. 2:**
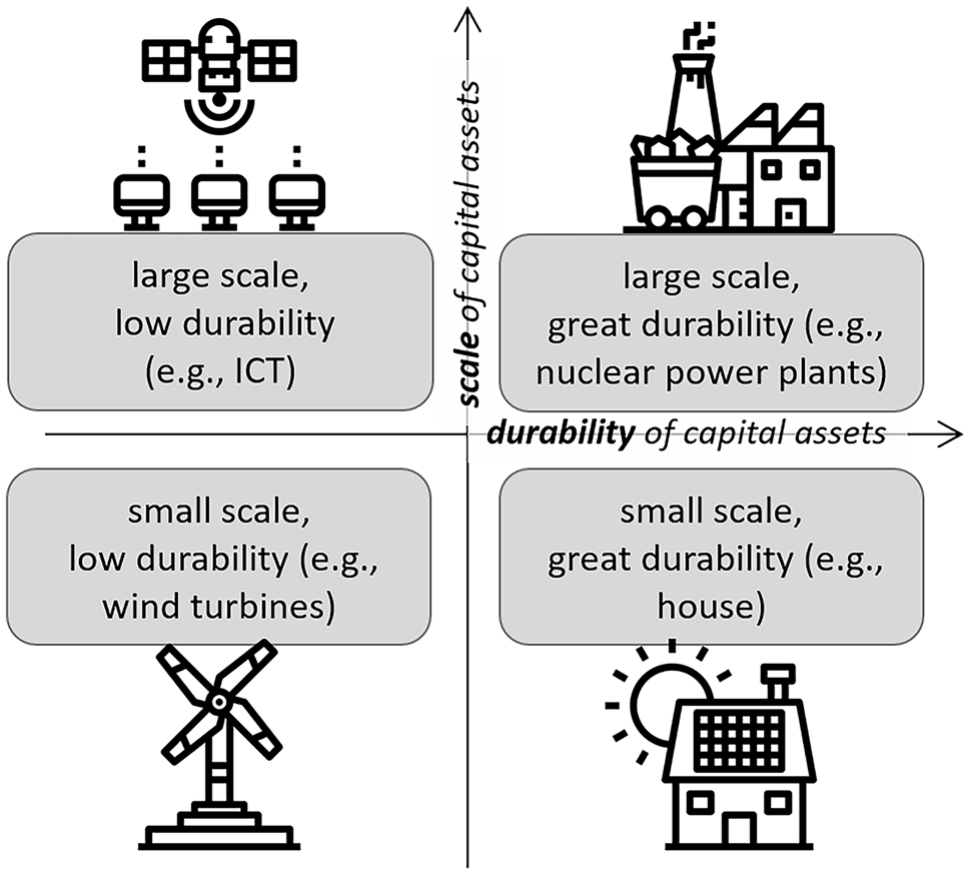
Sketch of scale and durability as defining properties of fixed capital assets, with information and communications technology (ICT) as an example of large-scale, low-durability assets. Please note that the provided examples must be understood in relation to one another and that appropriate examples may change over time. Concept from Harvey (1982), all icons by Payungkead (https://www.flaticon.com/authors/payungkead)

In a capitalist economy, fixed capital assets^1^ play a crucial role in locking-in specific forms of provisioning. Fixed capital can be distinguished according to its *scale* and *durability* (Fig. 2; Harvey 1982). These two characteristics are closely linked to typical patterns of investment, ownership, appropriation, and profit in provisioning systems. The larger the scale of the investment, the more a significant concentration of capital and/or state support and bank lending is required. Accordingly, actors and institutions involved differ by scale of the investment. The durability of fixed capital (measured as lifecycle or turnover time) depends on its material and socio-economic properties. A material asset has a limited physical lifetime but its use value may decline before its physical functionality does, due to devaluation associated, for example, with technological or political change or with shifts in preferences and practices.

The fossil energy system tends to require large-scale fixed capital of great durability, that is, large initial investments enabled by concentration of capital and/or access to loans and communal or state coverage of expenses. The lifecycle or turnover time of this fixed capital is generally long. Because of lock-in effects (Seto et al. 2016), significant future investment for maintenance must be anticipated. The characteristics of these assets support ownership by large-scale, complex, and often multinational private corporations. Large public monopolies, operating according to the same principle of maximizing yield, also exist.

Outside of direct ownership but nonetheless highly influential for fixed assets in provisioning systems are financial institutions and their specific logic of accumulation. For financial institutions seeking safe and long-term rent-generating assets, the material structures of the fossil energy system are attractive, because of their lock-in effects, their longevity, and the protection from competition they afford.^2^ Since the Paris agreement was reached, 33 banks have invested almost 2 trillion US dollars in fossil-fuel industries (Rainforest Action Network et al. 2019) and in 2018 alone, the same amount was invested in the existing global energy system (IEA 2019b). Notably, electricity utilities have systematically continued to invest in fossil-fueled generation (Alova 2020). The quasi-monopolies in this area of the economy have remained intact with extraction, processing, and distribution of fossil-fuel energy carriers representing highly concentrated processes, monopolized by a few large multinationals. In 2019, nine out of the world’s ten largest companies were part of the fossil energy system: six petroleum providers (Sinopec Group, Royal Dutch Shell, China National Petroleum, Saudi Aramco, BP, Exxon Mobile), two automobile manufacturers (Volkswagen, Toyota Motor), and one electricity company (State Grid Corporation of China) (Fortune 2019).

In the political economy of provisioning systems, we find “who sets the terms of energy transition and how, whose interests are served as a result and how relations of power […] shape the adoption of one energy pathway over another” (Power et al. 2016, p. 11). The longer the energy system has persisted in a given configuration, the more established the dominant power relations, and the more difficult it is to affect change (Carroll et al. 2018): “The stronger the vested interests—in particular incumbent energy interests—are, the more likely they are to have their interests represented with political decision-makers in the government, in the parliament, in the bureaucracy, as well as in the media, in academia, etc.” (Moe 2017, p. 347).

Comprehensive analyses of the configurations of these actors and the influence they wield need to consider the resources and physical infrastructures, buildings, and machineries on which the fossil energy system not only depends but also which in turn act to perpetuate this system.

The relationship between capitalist regulation and material stocks is a case-in-point for considering any provisioning system in integrated political-economic and socio-metabolic terms. This makes it possible to better appreciate how the current form of that system is stabilized and hence identify the obstacles to its transformation.

## (Re)shaping provisioning systems: phases and their possibilities for intervention

The social metabolism and the political economy of provisioning systems coalesce into shifting patterns of material stocks and flows, as well as actors and institutions. These shifts mark the beginnings and ends of loosely identifiable phases. Sketching out these phases elucidates the different processes that occur in the (re)production of any provisioning system and the potential entry points for sustainability transformations.

The (re)production of provisioning systems inevitably requires socio-metabolic and political-economic groundwork, investment and construction, operation, maintenance or replacement, and ultimately dissolution. In the following, we conceptually explore these phases, using examples from the fossil energy system. In this globally dominant energy system, the use of electricity requires generation in a power plant, for which coal deposits must be explored, mines excavated, transport networks built, power plants newly constructed, operated, and maintained, transmission networks upgraded, and old power plants decommissioned. Each phase is associated with specific changes in material stocks shaping the metabolic corridor and hence the present and future environmental impact of the provisioning system. In each phase, the trajectory of the system is shaped. Whether each phase leads into the next or not can be decisive in either reinforcing or challenging existing power relations. Many of the biophysical changes associated with land use change or resource extraction, with resource use and the generation of wastes and emissions are irreversible and future action must occur under the correspondingly changed conditions. In a generalized and simplified manner, we translate this constriction of the remaining option space into a narrowing of the socio-metabolic corridor for a particular provisioning system.

**Fig. 3:**
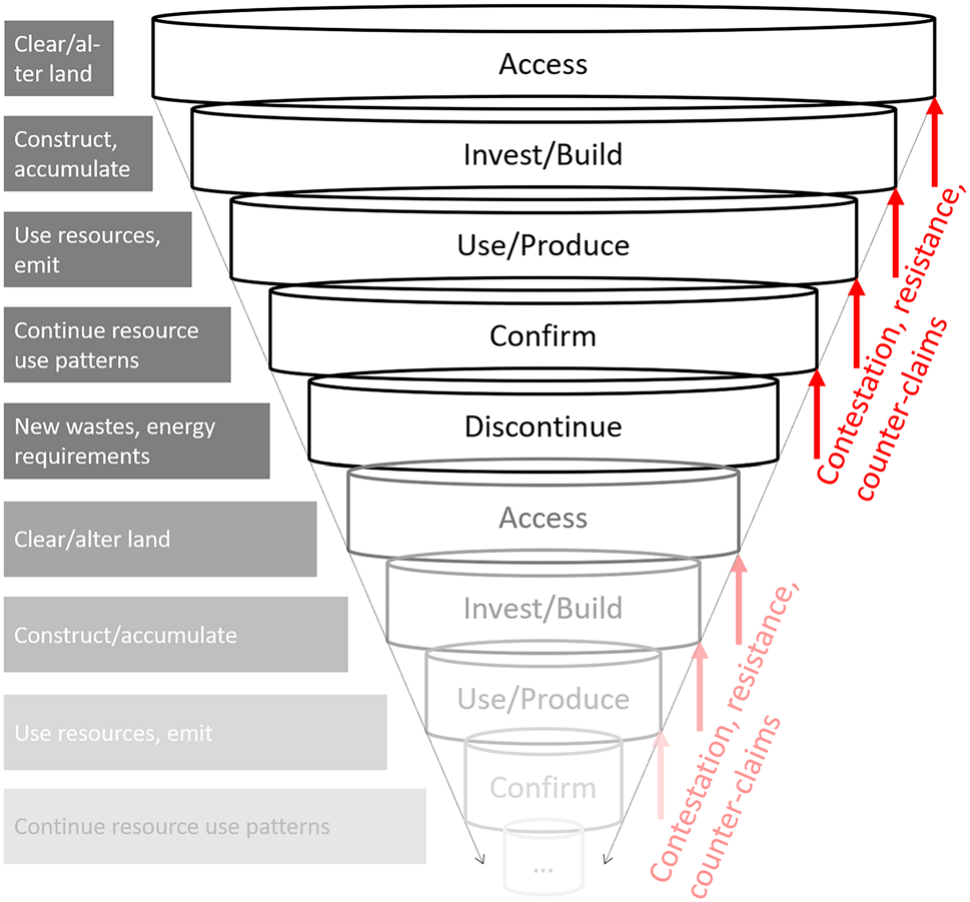
The spiraling constriction of the socio-metabolic corridor, described in “Phase 1: socio-metabolic and political-economic groundwork”–“Phase 5: obsolescence and dismantling” of this article. During five generalized phases of provisioning system development—(1) groundwork, (2) investment and construction, (3) operation, (4) maintenance, and (5) dissolution—new configurations are produced through access, investing and building, using and producing, confirming, and discontinuing. How they are produced socio-metabolically is associated with a constriction of the socio-metabolic corridor. Contestation, resistance, and counter-claims can intervene into this spiral at any point, pushing back again the prescribed progression. Although any discontinuation might be associated with a renewed implementation of the system elsewhere, this has to occur within a more strongly constricted metabolic corridor: as Germany plans to phase-out coal, new coal-fired power plants are being constructed around the world but will have to operate on globally reduced supplies of coal, for example

Our analytical focus on provisioning systems is partially motivated by the need to identify possible points of intervention into the inherent unsustainability of these systems’ current operations. Therefore, in the following delineation of the phases in the (re)production of provisioning systems, we are concerned with which counter-claims are made when and what potential they—if successful—have to enforce greater justice and sustainability (Fig. 3). Conflicts play an important role in challenging existing power relations, opening up potentials for alternative investments or denying fossil energy extraction and infrastructure (Pichler 2016; Temper et al. 2018).

### Phase 1: socio-metabolic and political-economic groundwork

Investments and the build-up of material stocks require socio-metabolic and political-economic framework conditions. Land may be cleared of people and of vegetation. By the 1990s, 2.5 million people in post-independence India had been displaced by coal mining (Fernandes 1998) with many more people affected as coal mines have continued to expand (Ahmad and Lahiri-Dutt 2006). The site ear-marked for exploitation is commonly connected to transport and utility infrastructure. In this phase, control over land is negotiated and/or (informally) established. Through processes of enclosure and territorialization, exclusive rights to land and resources are defined and claimed (Altvater and Mahnkopf 1999; Brad et al. 2015), often dispossessing local users from their territories in the process.

The groundwork cuts across policy arenas, encourages and dissuades the involvement of actors through the adaptation of taxation schemes, the introduction of subsidies, and the empowerment to levy rents (Deneault et al. 2012). Far-reaching changes to legislation may be spurred by the requirements of one specific infrastructure project and affect labor rights and working conditions, environmental protection, or energy and raw material prices (Agrawal and Ostrom 2001). Forms of exemption from existing legislation may also be introduced, in special economic zones and with the (unintentional) removal of bureaucratic hurdles, translating into additional gains of capital (Vandergeest 1996; Levien 2011; Cai and Aoyama 2018). Government activities may support access to workforce at the site, connection to utilities and transport mains, and the ‘management’ of protest and conflict (Janus 2012; Aguilar-Stoen 2016; Pye 2017). Capital invested in all manner of lobbying activities influences how the groundwork is laid. Research can, at this stage, legitimize or question the planned project in terms of its expected environmental and social impact, the assessment of risk or the general approach to (local) development (Finley-Brook et al. 2018).

Changed material constellations can induce further political-economic responses. A tool to politically integrate Brazil (Rosenbaum and Tyler 1971), the Trans-Amazonian Highway was materially expressed as changing material stocks (road, settlements) and flows (transport of people and goods) as well as land-use changes along its entire route. Some of these material changes then, in turn, ‘necessitated’ economic changes (e.g. of land prices) and legal changes (e.g. in land zoning) (Mäki et al. 2001; Fraser 2014).

At this stage, land conflicts are powerful points of intervention (Ramos Suárez and Pérez 2018). The local population may be able to resist land clearing and/or the site can be reclaimed through blockades. Legal claims, including (indigenous) land rights, environmental impact, cultural heritage may be made to deter the project. NGOs and even individual private actors may also—despite the relative lack in capital—engage in successful lobbying or information and public opinion campaigns. Overarching policy programs such as commitment to the Sustainable Development Goals, coal or nuclear phase-outs may additionally facilitate counter-claims to unsustainable provisioning systems.

In material terms, (some of) the damage has not yet been done in this phase. The socio-metabolic corridor has not yet been constricted. The local population, however, may already have been enticed or coerced into leaving their existing forms of subsistence for the transition to an industrialized metabolism. In some cases, the control over land serves a purpose in itself (e.g., to appropriate subsidies, obtain loans, speculate on future increases in land values), without the aim of ever actually using the land (McCarthy et al. 2012). In this sense, interventions in this phase are highly important to avoid the legacy effects that even initial, possibly symbolic changes can have. Access to land is the necessary precondition for the accumulation of stocks to occur so that (re)gaining control over land constitutes a very important lever.

### Phase 2: investment and construction

In this phase, material stocks are built up and their spatio-temporal configuration is determined, often quite literally set in stone or cast in iron. Especially for those fixed assets of large-scale and great durability (see “Capital, actors, and power relations: political-economic perspectives on provisioning”), the interventions made now are committed to in the long term. For example, draining an area of water so that foundations for a building may be built or that a mine may be excavated constitutes a commitment to keeping the ground water out in the future as well; sealing the soil requires a commitment to maintaining that seal if water and/or successions of plants are not to break through the surface, compromising intended use. Construction usually also requires high auxiliary resource flows, including energy and water as inputs and wastes and emissions as outputs.

These material changes require significant financial investment upfront and simultaneously create opportunities for further investment. Manifold capital sinks, and not their material outcomes, often constitute the prime motivation for engagement in infrastructure projects. The Tennessee Valley Authority, a US-American power utility, was established at the beginning of Roosevelt’s first term as US president to promote economic growth in an area especially hard-hit by the Great Depression. It resulted in the largest hydroelectric infrastructure project of its time, additionally generating electricity from nuclear and fossil fuels for a large portion of the US southeast (Schulman 1994).

Even without an immediate ambition to proceed to the operation phase, investment and construction may be attractive to capital, marking a claim to a territory and/or a provisioning system. Incentives include avoiding competition, tax advantages and exemptions, as well as subsidies (especially in the energy system) (Mitchell 2011). In the early 2010s, China, for example, had installed twice the wind power capacity of the USA but generated less electricity from this source than the USA (Moe 2017, p. 362). “It appears that China's policy approach to renewable energies has been to place priority first on developing its renewable manufacturing industry and only second on the generation of renewable energy itself” (Zhang et al. 2013, p. 349).

In this phase, actors providing the investment and executing the construction become increasingly influential, although state actors are required to maintain the necessary framework conditions. These can include, but are not limited to, protecting the investment through policing of the site and facilitating on-site labor. Labor intensity during this phase is high, and new (collective) actors such as workers and possibly trade unions become relevant. However, as the demand in construction tends to be for very cheap, temporary labor, the unionization rate can be very low (Barber 2016).

So-called build-operate-transfer initiatives have become fairly common for large-scale investments, especially within energy systems (Bakatjan et al. 2003): the investor receives a concession—usually from the government, making it a form of public–private partnership—to build and operate the project for a fixed time, during which the investor is entitled to all profits from operation. At the end of the run-time, the project is transferred to the contracting actor at no (additional) cost. These and similar forms of investment introduce both a shift of dominant actors in the lifetime of the project and a mix of actors in the operation and use phase. It leaves governments locked-in in a type of provisioning for which they are not directly accountable, because they outsourced the entire planning, construction, and establishing operational phase.

Interventions in the investment phase can re-open the discussion on what to do with the cleared land: can it be returned to the people or to nature? Does the previous population want to come back? Does the connection to auxiliary infrastructure (e.g., electricity, sewage) constitute a point of no return? Blockades of the construction site are a common tool in this phase and legal claims—including for impact assessments of the planned construction—may continue to be feasible. Labor and wage struggles can now—due to the involvement of the construction industry—play a more important role. Labor legislation and tax schemes or reforms can enable or hinder implementation.

### Phase 3: operation and use

During operation and use, the constructed stocks require and mediate resource throughput, including materials, energy, and water, and resulting wastes and emissions. A thermal power plant consumes coal and water, energy in various forms (including electricity for lighting, fossil fuels in transport), and emits ash and gases, especially CO_2_. These flows not only dwarf the amount of material of which the power plant consists but also account for the strongest environmental impacts during operation.

The distribution effects of the built stock manifests itself during this phase. Local pollution from emissions tends to be highest in close proximity to a facility, such as a power plant. The local population is not only exposed to this pollution but also tends to consume less (or none) of the goods or services produced: the marginalized, low-income communities around power plants, for example, can often not afford to consume the electricity whose generation diminishes local air quality (Pastor et al. 2010, 2013; Kopas et al. 2020). The goods or services that the project now generates (usually for final demand) is commonly viewed as its reason for being, although we have seen that up until and throughout this phase, capitalist accumulation and regulation often more decisively influence decision-making. The mining and processing of British coal, for example, were only very gradually modernized technologically as long as labor could be squeezed as a means of protecting profits and competitiveness (Turnheim and Geels 2012).

To generate surplus—possibly the dominant concern in this phase—the provisioning system in question must generate an output that can be sold, either to final consumption or as an intermediate input. The material stocks that make up the provisioning system can facilitate or hinder this process. The fossil-fueled electricity system, for example, is shaped by the main grid through which distribution of electricity from (mostly) large-scale power plants to final consumers is achieved. Access to this main grid has a gate-keeper function, potentially preventing the feed-in of renewable energy and excluding those not connected to it from consumption. This often prevents small-scale, decentralized renewable electricity from reaching remote consumers (Wang 2010).

Interventions during operation tend to center on halting specific processes, possibly with significant secondary effects throughout the provisioning system. With both production and consumption ongoing, much damage may already have been done, and lock-in is amplified. Enforced dependencies on the product or service sold protect the intended durability, especially of large-scale capital fixes. Nonetheless, by blocking sites of fossil energy carrier extraction (such as coal mines) or combustion (such as power plants), activists around the world have drawn attention to the socio-ecological impacts of the fossil energy system (Veltmeyer and Bowles 2014; Temper et al. 2015; Riffo 2017; Temper 2019). In some cases, activists have successfully carried legal proceedings against pollution stemming from operation and hazardous to human health. However, even such verdicts do not necessarily lead to operations ceasing: for example, following the victorious legal claims brought against the thermoelectric complex Parnaíba in Maranhao State, Brazil, the affected communities were to be resettled (EJOLT 2016). A court in Italy ruled that carcinogenic emissions made the further operation of steel mills in Taranto illegal; the government stepped in and was able to sell the bankrupt operator by offering the new parent company legal immunity (Barca and Leonardi 2016).

In Taranto and in much of the activism against the fossil energy system, environmental and labor struggles coalesce into joint interventions, often driven by working conditions and wages as well as issues of pollution. Where part of the surplus attained (amongst other things by keeping wages low) flows to the state through taxes, the government—in addition to the functions it may take on as outlined previously—plays an additional role in the capitalization of a provisioning system. During operation and use of material stocks, provisioning systems actively link production and consumption and new entry points for resistance and sustainability interventions open up. Forms of protest can emerge that include consumer boycotts at the household level alongside corporate- or government-level measures such as the termination or alteration of delivery contracts or remunicipalization (Becker et al. 2016). Divestment strategies, away from fossil-fuel-based production and corporations, also tend to take effect during operation (Healy et al. 2019).

Even if they are successful, these interventions are faced with the legacy of what has been constructed and used, and partially engrained into wider production and consumption patterns. The metabolic corridor within which sustainability transformations must occur has been severely restricted. The degree to which the amassed stocks can be flexibly used is important for the leeway to alter future socio-metabolic patterns. The greater the accumulation of material stocks at sprawling, mono-functional sites (such as coal mines and power plants but also strip malls on the urban fringes), the more difficult it becomes to integrate these stocks into sustainability transformations (Kincaid 2000). From Essen’s *Zeche Zollverein* (the German coal mine complex now part of the UNESCO World Heritage) to Chernobyl’s growing dark tourism, even some of the most clearly and disastrously mono-functional infrastructures are being culturally re-imagined in a manner that may be compatible with sustainability transformations. But given their construction and location, the majority of mines and power plants could only be alternatively used at high material and energy costs. The inflexibility of such large-scale assets of great durability constitutes an obstacle to sustainability transformations.

### Phase 4: maintenance and follow-up investment

Maintenance of and follow-up investment in a provisioning system often represents the affirmation of the original intervention, in material terms as well as in re-asserting the power relations thereby established. Maintenance can be required to allow the system and its components to continue functioning and generating surplus or for improvements, increasing productive capacity and surplus. This need not only be in response to deterioration or technological advances but could also occur in reaction to rising wages or improved labor laws making greater automation more attractive, for example.

The framework conditions spurring maintenance investment include the assessed residual value of the component or system and the expected follow-up investment (Harvey 1982), and also the value of linked infrastructure, questions of ownership (see “Phase 2: investment and construction”.), and operational function. This function does not have to correspond to the output that is delivered to final demand. It may be necessary to maintain an idle power plant even without a realistic option of powering up to mark the claim to land, because of the deterring effect of expected costs of demolition or because of contractual obligations to maintain the structure. In general, anything that is maintained still has some type of function for someone with decision-making power.

From the point of view of transformation, this phase may be crucial because, especially for large-scale investments of great durability, it represents a new moment of decision-making. Powerful points of intervention in this phase comprise divestment strategies or phase-out programs, as exemplified in the coal or nuclear industry (Renn and Marshall 2016; Ayling and Gunningham 2017; Johnstone and Hielscher 2017). The protests against the maintenance of coal infrastructure in Germany (‘*Ende Gelände*’; German, for “end of terrain”, a slogan signaling a blockade of any further land take of the open pit mine) have shown the politicizing potential that interventions into configurations dominated by economically powerful corporations (such as Germany’s second-largest electricity provider, the RWE AG) can have (Sander 2017). In a conflict executed by the police, RWE has sought to assert its claim to the Hambach Forest, a small wooded area in western Germany hosting deposits of lignite. In the face of anthropogenic climate change and biodiversity and habitat loss, activists have, for approximately a decade, sought to protect the area. RWE has planned to expand its open pit mine—currently already the largest in Europe—into this area to supply its thermal power plants with coal. The company’s argument is that this is necessary for the security of supply (Hajek 2018), evoking the electricity system’s reason for being. But even mainstream media are questioning whether—from a perspective of electricity supply—there are no other ways in which the same amount of electricity could be generated, especially in a country in which the government propagates the phasing-out of coal (Reuters 2019). This, however, would certainly not serve to safeguard the existing system in the face of competing claims as the maintenance investments would set out to do.

### Phase 5: obsolescence and dismantling

This phase can coincide with a change in the actors involved but more often involves the same actors diverting their investments, as exemplified by the role that big fossil energy corporations play in developing renewable energy. Efforts of BP p.l.c., formerly known as “British Petroleum”, to reinterpret its company name as “Beyond Petroleum”, represent an interesting case. It highlights both the ambition of BP to claim a share in this new market, as well as its greenwashing efforts in the face of its responsibility for the catastrophic oil spill in the Gulf of Mexico in 2010 (Cherry and Sneirson 2010; Kennedy 2010; Muralidharan et al. 2011). Dismantling will be eventually required to clear the occupied land and to free the components and materials for re-sale (e.g. through technology transfers or to recycling). Dismantling can thus become an option for generating surplus beyond the functional use of an element of the provisioning system (UNEP 2012; Schindler and Demaria 2019).

The cessation of intervention at the structure’s site is not equivalent to a return to the original state (“Phase 1: socio-metabolic and political-economic groundwork”.). The legacies of material stocks may extend for decades, centuries, and even millennia beyond the end of their use phases (Fentiman and Zabbey 2015; Winiwarter et al. 2016). Settlements formed around production sites persist even when that production does not (e.g., mining towns) and may continue to experience the environmental and health impacts from production (Lutz et al. 2013; Boyles et al. 2017). Claims to land are not reversed when a project is discontinued (or never begun e.g., land grabbing). The legacies of provisioning systems can also be related to the emissions and wastes generated during their operation: CO_2_ in the atmosphere will outlive the thermal power plant that emitted it, radioactive waste (including the power plant itself) must be managed when nuclear power plants are shut down. The longer a system was in operation, the more wastes and emissions will have accumulated. Wastes are often transported elsewhere for dismantling or disposal, with significant impacts on local economies and ecosystems (e.g., Demaria 2010) adding an additional layer to the spatial re-configuration associated with the discontinuation of specific forms of provisioning. Whether this distribution is planned and controlled (wastes are collected, treated and/or discharged to managed sites) or accidental side-effects (wastes are spilled during operation or transport), it will shape the discharge sites, possibly making them toxic and uninhabitable. Interventions during this phase tend to focus on (re)claiming land and exposing legacies. These may also take the form of corrective justice claims (Liszka 2010). Both in the material and the figurative sense, industrial wastelands are evoked in information campaigns as important references for interventions in the earlier phases of similar projects.

## Challenges and opportunities for the transformation of provisioning systems

When it comes to understanding and meeting the sustainability challenge posed by existing provisioning systems, their consideration along interrelated socio-metabolic and political-economic dimensions reveals not only barriers to transformation but also opportunities for intervention. We argue that the analysis of different phases in the (re)production of provisioning systems is a useful framework to structure analyses aimed at identifying such barriers and opportunities.

From laying the groundwork to construction, operation and use, maintenance, and (partial) dismantling and replacement, capital has been sunk into existing provisioning systems (Moe 2017, p. 346). This implies that strong vested interests and profitability expectations oppose far-reaching transformations of these systems. At the same time, material stocks may, through their legacies, be prohibitive for transformative change, or at least greatly reduce the speed of transformations (Sovacool 2016).

The capitalist regulation of provisioning systems has consequences for their techno-material functionality, shaping the resource use patterns within which they exist. Applied to their current form, provisioning systems have been built in response to the demands of capital at least to the same extent (if not more strongly so) as to final demand for the goods and services and the contribution to societal wellbeing they provide. While the use of fossil energy undoubtedly made comforting and even life-sustaining services and commodities accessible to parts of the population, fossil energy has also provided the capitalist economic system with crucial opportunities for surplus generation and absorption (Yergin 2012). The fossil energy system emerged from specific state and corporate interests, not from the ambition of low-impact generation and just distribution of energy services for social wellbeing (Mitchell 2011; Malm 2016). Legacy effects, techno-material and economic lock-ins consistently push other benefits of the systems—including their ability to absorb and produce surplus, to control people and territory—to the foreground. The stocks currently being built up not only constrict our metabolic corridor but also continuously lock-in actors and institutions and the power relations that bind them.

Given the strong inertia and power relations built into provisioning systems, individual consumer choices, market-driven or mere technological solutions are unlikely to bring about transformative change. Instead, a systemic transformation requires multiple and collective points of intervention that disrupt the socio-metabolic as well as the financial flows and challenge vested interests. In “(Re)shaping provisioning systems: phases and their possibilities for intervention”, we showed that different phases in the build-up of the fossil energy system and its components provide specific opportunities for intervention. The most immediate of these relate to conflicts that challenge the further *expansion* of the fossil energy system (Martinez-Alier 2014). These conflicts comprise, for example, resistance against the further extraction of fossil energy carriers (e.g., mining, fracking) and the respective fossil infrastructure development (Özkaynak et al. 2012; Veltmeyer and Bowles 2014; Riffo 2017). From an integrated socio-metabolic and political-economic perspective, recent struggles for the ‘municipalization’ of energy supply—and especially of renewable energy development—may be examples for transformative entry points (Kunze and Becker 2015; Moss et al. 2015; Haas 2019). In (re)municipalization, the techno-material expansion of renewable energy can be simultaneously directed against private control of energy supply and towards public municipal control over energy utilities. In the related struggles, beyond questioning state ownership, proposals for participatory public energy utilities are often developed (Moss et al. 2015). As scientific and political pressure for decarbonization accelerate, points of intervention are increasingly geared towards the *divestment and phase-out* of existing fossil energy system, for example, coal-fired power plants, oil refineries, etc. (David 2018; Healy et al. 2019). Together, these conflicts are frequently labeled as environmental justice or socio-ecological distribution conflicts (Temper et al. 2018).

Applied to the fossil energy system at large and electricity supply in particular, an integrated political-economic and socio-metabolic conceptualization of provisioning systems advances our understanding of these systems in two essential ways: first, it opens the door to a more holistic perspective on how current provisioning systems function and on how they are sustained, even in the face of severe contestation, resistance, and competing claims. Second, it allows us to conceptually embed provisioning systems within their historical context, simultaneously making it possible to conceive of different forms of provisioning under (radically) altered conditions.

The provisioning process for any good or service could be organized and realized in a number of ways other than the one that is in place today. Although there is an undeniable tendency for capitalist provisioning to take on similar forms across time and space, differences continue to exist in key areas including provisioning of energy, food, and shelter. Decentralized and locally administered production of electricity, subsistence farming and cooperative agriculture, and social housing initiatives implement alternative forms of provisioning, within the constraints of the capitalist system. The research already done on such initiatives could be expanded to better understand the political-economic and socio-metabolic operating space to which they can lay claim. It is not provisioning in and of itself that is problematic and conflict-prone but the specific form of its realization under capitalist regulation. This means that provisioning could function quite differently than it does in the systems available to us today.

## Footnotes

1 Fixed capital also takes the form of intangible assets such as patents, brands, standing, goodwill, etc. In addition to the tangible assets on which we focus, these intangible assets are very important in understanding why provisioning systems function as they do.

2 The electric grid, for example, is often described as a ‘natural’ monopoly, because economies of scale are so large that building a second, competing system is not viable (Sharkey 1982).
